# AZA Toxin Profiles by LC-HRMS in Shellfish from Šibenik Bay: AZA-2 Predominant Analog

**DOI:** 10.3390/molecules31010060

**Published:** 2025-12-23

**Authors:** Antonija Bulić, Ivana Pezelj, Ivana Ujević, Tanja Bogdanović, Stjepan Orhanović

**Affiliations:** 1 Institute of Oceanography and Fisheries, Šetalište Ivana Meštrovića 63, 21000 Split, Croatia; bulic@izor.hr (A.B.); ujevic@izor.hr (I.U.); 2 Faculty of Science, University of Split, Ruđera Boškovića 33, 21000 Split, Croatia; izaper@pmfst.hr; 3 Croatian Veterinary Institute, Poljička Cesta 33, 21000 Split, Croatia; t.bogdanovic.vzs@veinst.hr

**Keywords:** LC-MS/MS, LC-QTOF, CID spectrum, Adriatic Sea, shellfish biotoxins

## Abstract

Azaspiracids (AZAs) are marine polyether biotoxins produced by dinoflagellates that accumulate in filter-feeding organisms and pose a threat to human health and seafood safety. This study presents the first comprehensive analysis of azaspiracid analogs in shellfish from the Adriatic Sea with the use of high-resolution mass spectrometry. AZA-2 was quantified in samples collected from Šibenik Bay between January and May 2024, with the highest concentrations observed in early January. In addition to AZA-2, several known analogs (AZA-6, AZA-9, AZA-10, AZA-19, AZA-41, and AZA-43) and a potentially new analog (*m*/*z* 884.4928) were also detected. The fragmentation patterns of this new analog indicate a structural similarity to AZA-19 with a possible double bond modification. Potential pitfalls regarding the misinterpretation of spectra derived from molecules containing ^13^C atoms were recognized and addressed. The presence of multiple analogs, some of which have high toxic potential, suggests that regulatory practice should consider including more than three analogs in the monitoring program.

## 1. Introduction

Azaspiracids (AZAs) are a group of marine polyether biotoxins first identified in 1995 following a case of poisoning in the Netherlands, in which people exhibited symptoms resembling diarrheic shellfish poisoning (DSP) following the consumption of mussels harvested in Ireland [1]. Initially, the toxin was named “Killary toxin”, but after its structural characterization, it was renamed azaspiracid-1 (AZA-1) to reflect its major structural features: a cyclic amino group, three spiro bonds, and a carboxylic acid group [2]. Different AZA toxin analogs were described in 1999 in samples from a toxic episode in Ireland in 1997 and named AZA-2 and AZA-3, followed by the discovery of numerous analogs with a common structural backbone modified by the introduction of double bonds and methyl, hydroxy, and carboxy substituents at characteristic positions [3,4]. The structures of some analogs have been confirmed with spectroscopy of nuclear magnetic resonance (NMR), while most structures are deduced or suggested using mass spectrometry. Currently, the total number of analogs appears to be 68. Despite the large number of identified analogs for AZA toxins, regulatory monitoring is focused on AZA-1, AZA-2, and AZA-3. Although some analogs with toxicity at the same scale as regulated analogs have been described, at present, the other isomers or analogs are not considered to contribute significantly to the overall toxicity associated with this toxin group. Some of these compounds are produced by phytoplankton and accumulate in shellfish, where they undergo structural changes as a result of shellfish metabolism [5]. Given their potential impact on human health and the shellfish farming industry, AZAs have become the subject of intense research and ongoing monitoring in various regions of the world, including the Adriatic Sea.

Initially, it was believed that heterotrophic dinoflagellates of the genus *Protoperidinium* produced the azaspiracids. However, it was found that they only accumulate these toxins by feeding on phototrophic dinoflagellates [6]. This was confirmed in 2007 when the phototrophic dinoflagellate *Azadinium spinosum* was found and isolated in the northern Atlantic Ocean. *A. spinosum* was subsequently shown to produce the toxins AZA-1, AZA-2, and AZA-33 [7]. In addition to these toxins, *A. spinosum* is also known to synthesize AZA-11, AZA-34, AZA-35, AZA-50, and AZA-51 [8]. After the discovery of *A. spinosum* in the late 2000s and early 2010s, it was confirmed that other dinoflagellates from the *Amphidomataceae* family also produce azaspiracids. Research soon showed that the genera *Azadinium* and *Amphidoma* are more widespread than initially recorded and have been detected in many coastal waters of Europe, Asia, and South America [7]. The dinoflagellate *Azadinium poporum* is a well-documented producer of AZAs, with different strains having different AZA profiles depending on their geographical origin. This species has been found to produce AZA-2 as the main analog in strains from Argentina and the Mediterranean, while strains from Chilean coastal waters predominantly synthesize AZA-11 [9]. Other analogs such as AZA-36, AZA-37, AZA-40, AZA-41, AZA-42, AZA-59, and AZA-62 have been identified in different strains, further illustrating the biosynthetic variability of this species [8]. Another closely related species, *Amphidoma languida*, has been identified as a producer of azaspiracids, in particular AZA-2, AZA-38, AZA-39, AZA-43, AZA-52, and AZA-53 [10,11,12]. A recently described species, *Azadinium dexteroporum*, has been shown to synthesize several AZAs, including epi-AZA-7, AZA-35, AZA-54, AZA-55, AZA-56, AZA-57, and AZA-58 [13,14,15]. A comprehensive compilation of AZAs produced by dinoflagellates from 2019 lists 26 variants, including two newly described variants, AZA-42 and AZA-62 [8]. It should be noted that due to the extremely small size of dinoflagellate cells, reliable detection methods must be used, which usually include PCR analysis of phytoplankton samples [16,17].

A number of studies focused on mechanisms of toxicity and have shown that AZAs can modulate the permeability of various ion channels (potassium, sodium, chloride, and calcium). However, it is not yet entirely clear whether they bind directly to target proteins or act via intermediates [4]. Gastrointestinal symptoms (diarrhea, nausea, vomiting, abdominal pain) may also occur due to changes in the intestinal glial system and a weakening of the integrity of the intestinal barrier [18]. AZAs have some effect on the shellfish feeding on AZA-producing dinoflagellates. Upon feeding of shellfish *Mytilus galloprovincialis* with culture of *A. dexteroporum*, changes noted in shellfish tissue included inhibition of phagocytosis activity of mussel hemocytes, damage to lysosomal membranes, thinned tubule walls in the digestive tissue, and slight genotoxic damage; however, most changes were reversible upon toxin removal [15].

The structural diversity of AZAs arises from modifications such as methylation and hydroxylation, which occur predominantly at the carboxyl end of the molecule [6]. However, structural variations extend beyond this region, including oxidation, reduction, carboxylation, and shortening of the carbon chain, which have been observed in analogs such as AZA-33, AZA-34, and AZA-35 [19]. Additionally, modifications in the nitrogen-containing I-ring contribute to this diversity. Most AZAs have a saturated I-ring that is methylated at the 39-position, resulting in a characteristic *m*/*z* 362 fragment when analyzed by mass spectrometry. In some cases, the I-ring is unsaturated (*m*/*z* 360) or demethylated (*m*/*z* 348) [8].

Apart from the structural diversity due to differences in biosynthetic pathways, the potential biotransformation in shellfish presents a major challenge for AZA research. Primary forms such as AZA-1 and AZA-2 are enzymatically transformed to produce hydroxylated or methylated derivatives with altered polarity, toxicity, and metabolic stability in marine organisms [20]. Biotransformation in shellfish was studied in experiments in which blue mussels were fed with AZA-1- and AZA-2-producing dinoflagellate *A. spinosum*. Rapid transformation of original toxins to their metabolites, AZA-3 to AZA-12, AZA-17, AZA-19, AZA-21, and AZA-23, was observed. Already after 6 h, metabolites reached 25% of total AZAs, and their content increased to over 50% after 24 h, with AZA-17 and AZA-19 detected as the most dominant transformed derivatives of AZA-1 and AZA-2, respectively [21]. Additionally, biotransformations of AZA-59 produced by *A. popurum* and AZA-38 and AZA-39 produced by *Am. languida* were studied in the feeding experiments with *Mytilus edulis.* Investigated toxins from both organisms underwent biotransformations like hydroxylation, carboxylation, and demethylation. Additionally, AZA-59 with the hydroxyl group at C3 was esterified to a significant amount with fatty acids [22,23]. Despite this structural diversity, routine monitoring programs focus only on the sum of AZA-1, AZA-2, and AZA-3 as regulatory markers for seafood safety. To present the results in accordance with the European legislation for permitted levels, azaspiracids AZA-1, AZA-2, and AZA-3 are expressed as µg AZA-1 eq./kg (µg AZA-1 equivalents/kg) by applying toxicity factors for AZA-1, 1; AZA-2, 1.8, and AZA-3, 1.4. The European Union and the United States have implemented a limit of 160 µg AZA eq/kg shellfish for the sum of AZA-1, AZA-2, and AZA-3 [24,25], as these are the most common analogs found in the human diet. However, emerging evidence suggests that other analogs, including AZA-6, AZA-17, and AZA-19, may also have significant toxic potential [21,26]. Understanding the distinct fragmentation patterns of AZAs is crucial for accurate toxin identification and monitoring, particularly in the context of regulations that focus on the most toxic and prevalent analogs [4,6]. Monitoring of mussels from the Adriatic Sea for biotoxins is carried out in several key locations, including the Šibenik area and surrounding waters. Azaspiracids have been detected so far in the Adriatic Sea only sporadically and at lower levels. AZA-2 was detected at concentrations slightly above the level of detection, reaching a maximum value of 3.5 μg/kg w.w. in the Bay of Neum (August 2017). AZA-2 was detected in the Makarska City Bay in one sample only, collected in July at a concentration of 3.8 μg/kg w.w. [27]. The first report of AZA-2 in mussels from the North Central Adriatic Sea is described by Bacchiocchi et al. (2015), even though reported only in trace amounts [28].

Our study aims to provide a temporal assessment of the appearance of AZA toxins in Šibenik Bay waters in January 2024. Another focus is the in-depth analysis of shellfish samples for the presence of various AZA analogs and the assessment of their contribution to the overall toxicity of the analyzed mussels. Through the use of high-resolution mass spectrometry, this research aims to provide a detailed examination of the fragmentation patterns of detected analogs and potentially identify previously uncharacterized azaspiracid analogs. The identified suite of azaspiracid analogs is intended to be used to eventually draw conclusions about the causative species responsible for producing the toxins.

## 2. Results

### 2.1. Quantitative Determination of the AZA 2 Content

**Figure 1:**
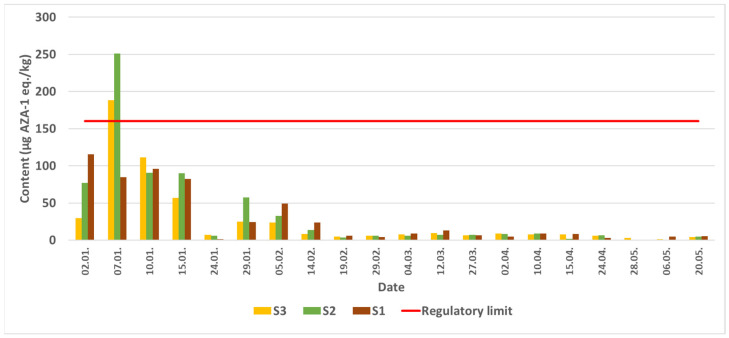
AZA-2 content in mussels from Šibenik bay from January to May 2024, determined by the LC-MS/MS, red S1, green S2, and orange S3.

Quantitative analysis of the mussel samples did not reveal the presence of AZA-1 and AZA-3 in any of the samples analyzed. AZA-2 was detected for the first time in samples from S1, S2, and S3 on 2 January 2024. The highest AZA-2 content was detected on 2 January 2024 at sampling site S1 and on 7 January 2024 at sampling sites S2 and S3, followed by a gradual decrease in AZA-2 levels until 20 May 2024. Latter no AZAs were detected in the samples from these sites (Figure 1).

### 2.2. AZA Toxin Profiles in Mussels from Šibenik Bay

Several analogs, in addition to the most dominant AZA-2, were detected using high-resolution mass spectrometry. A total of 8 analogs were detected separately, differing in precursor mass, fragmentation pattern, and retention time (RT). After analysis of their fragmentation patterns and comparison with data from the literature [2,8,10,11,14,19,29,30,31,32,33,34], seven of them were recognized as AZA-2, AZA-6, AZA-9, AZA-10, AZA-19, AZA-41, and AZA-43. A comparison of their exact and calculated masses, determined from a representative sample collected at station S2 on 2 January 2024, is shown in Table 1 along with their RT [8,28].

Fragmentation of two analogs set to *m*/*z* 856.5 and 858.5 resulted in spectra containing clusters of three peaks differing by one *m*/*z*, indicating fragmentation of a compound containing two ^13^C atoms. These spectra were analyzed and assigned to the known analogs. 

The analog with fragmentation set to a precursor *m*/*z* of 884.5 showed a fragmentation pattern that, to our knowledge, has not yet been recorded. The analog we recorded, with RT 3.89 min, had an exact *m*/*z* of 884.4928. Its fragments included 866.4739 (precursor water loss), 840.4946 (precursor -CO_2_), 822.4769, 804.4679, 786.4602 (precursor-1-3H_2_O, -CO_2_), group 2 fragments starting at 700.3698, decarboxylated at 656.3778, followed by one and two water losses at 638.3677 and 620.3558, respectively. In group 3, fragment 490.2801 was detected with low intensity, while 446.2908, the product of its decarboxylation, was clearly observed. Group 4 and 5 ions, 360.2521 and 260.1644, were detected, with the group 4 ion followed by a signal at 342.2416 due to the loss of one water. The group 6 ion with an *m*/*z* of 166.1220 was also detected with low intensity.

**Figure 2:**
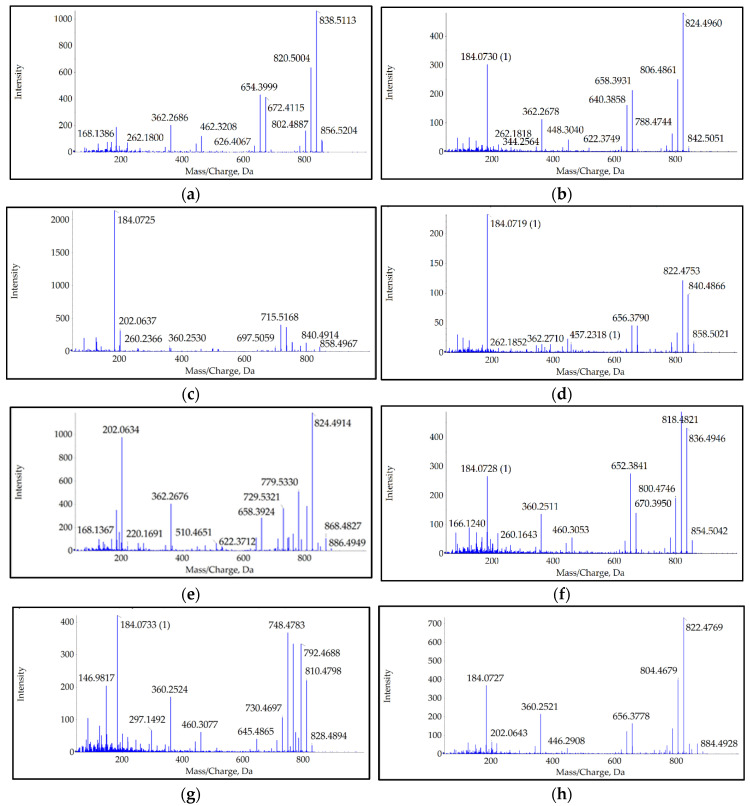
Mass spectra acquired by the SRM^HR^ method: (**a**) AZA-2, (**b**) AZA-6, (**c**) AZA-9, (**d**) AZA-10, (**e**) AZA-19, (**f**) AZA-41, (**g**) AZA-43, and (**h**) novel AZA 884.

The characteristic spectra of all analogs are presented in Figure 2. Due to the limited size of the images in the main text, presenting all relevant ions in a legible size would result in a cluttered figure. Therefore, spectra with larger images are also provided in a supplement Figure S1–S8, along with a table containing the *m*/*z* of detected fragment ions, their elemental composition, and the deviation of detected ions from theoretical values (Table S1).

### 2.3. Geographical and Temporal Dynamics of AZA Analogs

Selected samples were analyzed in detail to define the toxin profiles at the beginning of the outbreak, as determined by the amount of AZA-2 detected with the triple-quadrupole instrument, and later, during the declining stages of the toxic episode, Table 2. All detected analogs were present in all samples characterized by high toxin levels (samples from 2 January, 7 January, and 10 January). In samples with low AZA-2 levels from 24 January 2024, no AZAs were detected with the QTOF instrument. In samples with slightly increased AZA-2 levels collected on 29 January, 5 February, and 14 February, along with AZA-2, AZA-6 was detected in all samples, while AZA-19 was detected in station S3 on all dates; in station S2, on 14 February; and in station S1, on 5 February and 14 February. AZA-41 was detected in station S2 on 29 January. Among samples with low AZA-2 content collected on 19 February, only the S1 sample also contained AZA-6 and AZA-19. In samples collected on later dates, AZA-6 was detected only on 10 April, while AZA-2 was not detected in any samples from 20 May or in samples from stations S2 and S3 from 6 May.

## 3. Discussion

The AZA-2 toxin was detected and quantified in samples from Šibenik Bay in January 2024, and its presence was followed until May, when no more toxin could be detected in shellfish samples. Until now, AZAs have been detected in the Adriatic Sea only in low amounts, and their presence has not affected the shellfish farming industry [27,28]. The toxic episode described in this study indicates that AZA outbreaks may become a real threat to human health and shellfish farming in the area. High-resolution mass spectrometry was used to establish the presence of other known analogs in addition to the regulated AZA-2. Eight different analogs were detected, seven of which were already known: AZA-2, AZA-6, AZA-9, AZA-10, AZA-19, AZA-41, and AZA-43. The eighth analog, which fragmented at a precursor set at 884.5, exhibited fragmentation spectra that, to our knowledge, could not be assigned to any known analog. The only analog previously recorded with such a precursor *m*/*z* was AZA-56, with an exact precursor *m*/*z* of 884.5535 [12] and fragments at 866.5413, 848.5266, 830.5161, and 812.5051, consistent with precursor water losses; group 2 fragments at 690.4212, 672.4069, and 654.4000; group 3 fragment at 462.3203; group 4 fragment at 362.2682; and group 5 fragment at 262.1797. Such mass spectra are not consistent with the spectra from our samples. Instead, all peaks we detected are similar to those of the AZA-19 analog but shifted by two Daltons to a lower *m*/*z*, indicating the introduction of a double bond in the smallest fragment region (rings H or I) of AZA-19 [30]. Among the large number of AZA analogs, some differ in *m*/*z* by two Daltons, resulting in possible misidentification of an analog with two ^13^C atoms as a molecule with a two Dalton larger *m*/*z*, as noted by Krock et al. 2014 [31]. Such a fragmentation spectrum resulting from ^13^C-containing molecules can be recognized by typical clusters of three peaks that differ by one *m*/*z*, a consequence of the distribution of ^13^C atoms in the fragments formed. In our case, AZA-41 (*m*/*z* 854.5) with two heavy C atoms fragmented in the window of AZA-2 (*m*/*z* 856.5), and heavy AZA-2 fragmented in the window of AZA-9 and AZA-10 (*m*/*z* 858.5). Their characteristic spectra, containing typical clusters of three peaks, are presented in Supplement Figure S9.

Among the detected analogs, only AZA-2, AZA-41, and AZA-43 are of phytoplanktonic origin; the other analogs are products of shellfish metabolism [6,8]. Since no causative dinoflagellate has been confirmed in water samples from the affected sites, the origin of the toxicity outbreak can only be speculated based on published data. Azaspiracids were first detected in the Adriatic Sea in 2015, with no causative phytoplankton species identified [28]. The only AZA producers detected so far in the Mediterranean Sea were *A. dexteroporum* in the Gulf of Naples and *A. poporum* in the waters of Corsica [14,35] and the Ionian Sea [36]. AZAs that have so far been detected as toxins produced by *A. dexteroporum* include AZA-35, AZA-54 to AZA-58, and 3-epiAZA-7, none of which were detected in our samples. Different strains of *A. poporum* at various geographical locations produce AZA-2, AZA-11, AZA-36, AZA-37, AZA-40 to AZA-42, AZA-59, and AZA-62 [8,35]. Among these, AZA-2 and AZA-41 were detected in our samples. AZA-43 has so far only been detected as a product of *Am. languida* [10], but it cannot be excluded that it may be produced by a strain of *A. poporum* or be a product of shellfish metabolism. Other analogs detected in this study could be formed by biotransformations of AZA-2 and AZA-41. AZA-19 could be formed from AZA-2 by C-22 Me oxidation and can be transformed to AZA-6 by C-22 decarboxylation. AZA-6 can be C-23 hydroxylated to form AZA-10, or C-3 hydroxylated to form AZA-9 [37]. Direct transformation of AZA-2 to AZA-19, its subsequent decarboxylation to AZA-6, followed by hydroxylation to AZA-9 and AZA-10, was already noted by Jauffrais et al. (2012), who studied biotransformation of azaspiracids in cultured mussels exposed to the toxin producer *A. spinosum* [21]. All toxins formed by shellfish transformation of AZA-2 were present in the high-toxicity samples, while in samples with less than higher AZA-2 content, the most prominent analog was AZA-6 and, to a lesser extent, AZA-19.

Attempts were made to quantitatively assess the contribution of detected analogs to the overall amount of toxins present in the shellfish sample. Areas of survey scan extracted ion chromatograms of detected analogs were determined and related to the same data for AZA-2 in Table 2. Although precise comparison is not possible due to their different molar responses, it is informative to note that in samples with high AZA-2 content, no analog is present in an amount exceeding 77% of AZA-2. In high-toxicity samples, the most abundant analogs besides AZA-2 were AZA-19, AZA-41, and AZA-43. In samples with low AZA-2 content, AZA-6 and, to a lesser extent, AZA-19 are present in amounts that in some cases exceed those of AZA-2. Both AZA-6 and AZA-19 are products of AZA-2 biotransformation. This difference in the occurrence of various analogs could reflect differences in the dynamics of toxin production, rates of bioaccumulation and depuration in shellfish, or the possibility that some toxins are present in lower quantities and fall below the detection limit earlier. Clarifying the dynamics of analog occurrence would require a method suitable for quantifying a range of different analogs.

Different analogs vary in their toxicity, so transformation can alter toxicity for humans. Since only AZA-1, AZA-2, and AZA-3 are monitored as directed, some level of toxicity could remain undetected due to transformation. The contribution of various analogs to total toxicity is an important and as yet unresolved issue. Assessing the contribution of individual toxins to overall toxicity is complicated by their different molar responses in the LC-MS/MS quantification method and their widely varying toxicity. Only AZA-1, AZA-2, and AZA-3 have commercially available standards regulated by authorities. Estimating the amount of other analogs using these standards is hampered by differences in the molar responses of either the parent ions or the fragments used to quantify the compounds in extracts. The relative response factors of AZA-4 to AZA-10 differ significantly from AZA-1 under both isocratic and gradient conditions, and this difference was even more pronounced for the hydroxylated analogs [26]. The toxicity of the analogs can be compared using several methods, adding complexity to the assessment of overall toxicity. Using the Jurkat T-lymphocyte cytotoxicity assay for AZAs 1–10, relative toxicities were determined based on EC50 values ranging from 0.1 to 3.1 mM. Notably, although AZA-2 was the most toxic, analogs AZA-6 and AZA-8 were found to be more toxic than AZA-3 and AZA-1, which are used by the European Food Safety Authority (EFSA) and Food and Drug Administration (FDA) to determine AZA toxicity [26]. In another study, the same test showed that analog AZA-34, with a shorter side chain at the carboxy end, had 5.5 times higher toxicity than AZA-1 [19]. The first in vivo study with intraperitoneal administration to mice showed toxicity equivalency factors (TEFs) of 1.0, 1.8, and 1.4 for AZA-1, AZA-2, and AZA-3, respectively, while another study reported different TEFs of 1.0, 0.6, and 0.5 for the same toxins [2,29,38]. Orally administered toxins showed lower potency, with TEFs of 1.0, 0.7, and 0.5, similar to more recent intraperitoneal tests [38]. Therefore, the overall toxicity and contribution of the detected analogs can only be roughly estimated. However, the presence of multiple analogs in our samples, as well as in most other samples, including some (such as AZA-6 in our samples) with significant toxic potential, suggests that assessing the toxic potential of azaspiracids may require quantifying more than three analogs, as has already been suggested [21,22,23].

## 4. Materials and Methods

### 4.1. Samples

**Figure 3:**
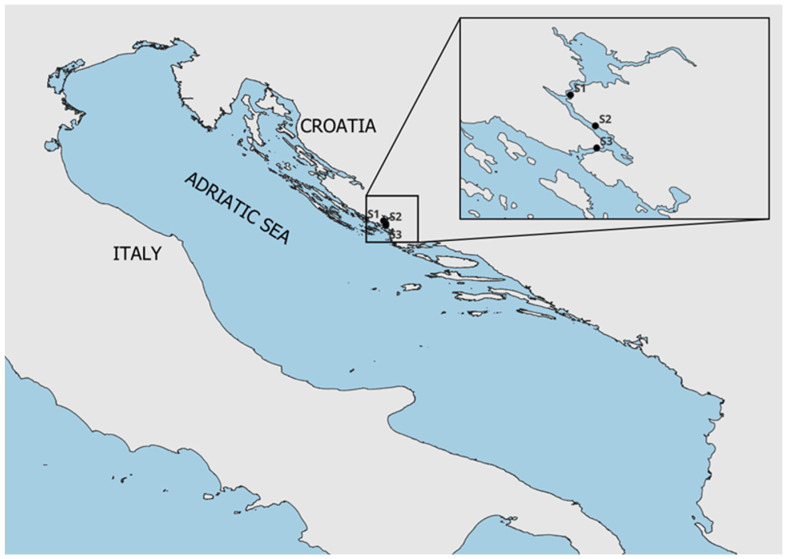
Sampling stations in Šibenik bay, S1—Strmica, S2—Šibenik, S3—St Antony’s Channel.

The presence of marine biotoxins in farmed mussels is continuously monitored at several locations along the Adriatic coast. Monitored biotoxins include azaspiracids, with analogs AZA-1, AZA-2, and AZA-3 included in the monitoring program. Samples were collected from shellfish farms at three locations near Šibenik (Figure 3).

The mussel samples (*Mytilus galloprovincialis*) were collected from January to May 2024. with a total of 60 samples collected during the study period. Samples were collected at regular weekly intervals, with one sample per site each week.

### 4.2. Chemical Analysis

#### 4.2.1. Sample Preparation for LC-MS/MS Analysis

Acetonitrile (ACN), methanol (MeOH) and water (H_2_O) were purchased from J.T. Baker-Avantor (Madrid, Spain) and were LC-MS grade. Formic acid (98–100% purity) and ammonium formate (≥99% purity) were purchased from Sigma-Aldrich (Madrid, Spain). Samples were prepared in compliance with the European Union (EU) standard operating procedure for the determination of lipophilic marine biotoxins in mollusks [39]. Approximately 150 g of wet soft tissue was separated per sample and homogenized with a blender (22,000 rpm, 3 min). From the resulting homogenate, 2.00 ± 0.05 g of tissue was weighed and extracted with 9 mL of methanol (MeOH). The mixture was vortexed for 2 min and centrifuged at 4500× *g* for 10 min. The supernatant was transferred to a clean tube, and the extraction was repeated by adding an additional 9 mL of MeOH to the pellet. The supernatant from the second extraction was combined with the first, and the final volume was adjusted to 20 mL with MeOH. The combined extract was then filtered through a 0.22 μm nylon syringe filter into vials for further analysis.

#### 4.2.2. Certified Reference Materials

Certified reference materials (CRMs) for lipophilic toxins were obtained from the Institute of Marine Sciences, National Research Council (Halifax, NS, Canada) for LC-MS/MS analysis. These included AZA-1, AZA-2, AZA-3, and freeze-dried mussel tissue (FDMT1) as reference material to verify method accuracy (accuracy value was 108.62%, required range 90–110%). To match the sample matrix, 0.35 g of FDMT1 was weighed, and 1.65 mL of water was added. The extraction procedure was then identical to that described for the samples under Section 4.2.1.

#### 4.2.3. Detection of Lipophilic Toxins Using the LC-MS/MS Method

The regulated AZAs were detected and quantified using the European Union (EU) validated standard operating procedure for the determination of lipophilic marine biotoxins in mollusks, based on a triple quadrupole instrument [39]. The instrument used was an LC (1100, Altium, Zagreb, Croatia) coupled to a triple quadrupole mass spectrometer (6410, Altium, Zagreb, Croatia) equipped with an electrospray interface. Chromatographic separation was performed on a C18 Poroshell 120 column (50 mm × 2.1 mm, particle size 2.7 μm, Altium, Zagreb, Croatia) coupled with a Poroshell 120 EC-C18 guard precolumn (5 mm × 2.1 mm, particle size 2.7 μm, Altium, Zagreb, Croatia).

The mobile phases were water (phase A) and 95% acetonitrile (ACN) (phase B), both containing 2 mM ammonium formate (AF) and 50 mM formic acid (FA). Before mixing with ACN, AF was dissolved in 1 mL of water. A linear elution gradient was applied, progressing from 10% to 80% phase B over 4 min, held for 2 min, reduced to 10% phase B over 0.5 min, and held for an additional 4.5 min to equilibrate the column for subsequent runs. The flow rate of the mobile phase was 0.3 mL/min, and the injection volume was 2 μL. The mass spectrometer operated in positive ionization mode for the detection of AZA-1, AZA-2, and AZA-3 with a spray voltage of 3.5 kV. The capillary temperature was maintained at 350 °C. Regulated AZAs were quantified by analyzing predetermined qualification and quantification fragments (dependent scan) listed in Table 3.

#### 4.2.4. Characterization of AZA Toxins Using High-Resolution Mass Spectrometry

Identification and characterization of analogs not included in the targeted QQQ method require the use of high-resolution mass spectrometry. An ultra-high-performance liquid chromatography (UHPLC) system (ExionLC AD series, Sciex, Framingham, MA, USA) was coupled to a high-resolution mass spectrometer (TripleTOF 6600+, Sciex, Framingham, MA, USA) equipped with a DuoSpray Ion Source operated in ESI mode and a Quadrupole Time-of-Flight mass analyzer controlled by Analyst TF 1.8.1 software. A Kinetex Core-Shell C18 column (100 mm × 2.1 mm, 2.6 µm, Phenomenex, Cheshire, UK) coupled to a SecurityGuard ULTRA Cartridge C18 (Phenomenex) was used with mobile phase A (H_2_O) and mobile phase B (ACN:H_2_O, 95:5), both containing 50 mM FA and 2 mM AF. The column temperature was 30 °C, and the injection volume was 5 µL. At the start (0 min), the mobile phase contained 15% phase B. During the first 2 min, the composition changed to 65% phase B. This composition was maintained for 9 min. At 10 min, the composition returned to its initial state of 15% phase B and remained constant until the end of the run at 13 min. The flow rate was 0.3 mL/min and remained constant throughout the run. Samples were first analyzed in positive ionization mode using the time-of-flight mass spectrometry (TOF-MS) method without fragmentation of precursor ions. The total ion chromatogram was then searched for all known AZA precursor ions available from the literature. The detected precursor ions were then analyzed with the Selected Reaction Monitoring High-Resolution (SRM^HR^) method and manually examined for typical AZA fragmentation patterns. The source conditions were ion spray voltage front (ISVF) = 5500 V; capillary temperature at 425 °C, GS1 at 40, GS2 at 50, and curtain gas at 30 (arbitrary units); collision energy (CE) = 10 eV for MS experiments and CE = 65 eV for MS/MS experiments; and declustering potential (DP) = 80 eV. The accumulation time for the TOF-MS method (MS experiment only) was 750 ms, resulting in a cycle time of 775 ms, while the accumulation time for the SRM^HR^ method was 100 ms for the TOF-MS scan and 150 ms for the MS/MS of selected ions. If necessary, to reduce cycle time, the precursor ions were grouped into three periods according to their RT as required, so that the cycle time for all experiments was approximately 2000 ms.

#### 4.2.5. Assessment of Contribution of the Various Analogs to the Total Toxicity

Since no standards are available for the different analogs detected, only their extracted ion chromatograms recorded in the survey scan could be used to roughly assess their contribution to the total toxin content. For this purpose, the area under the peaks in the extracted ion chromatograms was determined and expressed as a percentage of the AZA-2 area. Although their response factors are not the same, data acquired in this way provide insight into the contribution of unregulated analogs to the total toxin content and could be used to trace toxin transformation at various stages of a toxic episode. 

## 5. Conclusions

AZA-2 was detected and quantified using a triple quadrupole mass spectrometer in mussel samples collected at three stations in Šibenik Bay at the beginning of January 2024. Its content was monitored until no more toxin could be detected in May 2024. Several other AZA analogs were detected in the same samples using a QqTOF high-resolution mass spectrometer (TripleTOF 6600+, Sciex, Framingham, MA, USA). In addition to the previously described analogs (AZA-2, AZA-6, AZA-9, AZA-10, AZA-19, AZA-41, and AZA-43), an analog with a precursor mass of 884.4928 and a previously unrecorded fragmentation pattern was detected. The fragments obtained suggest similarity to AZA-19, as all fragments correspond to AZA-19 but are smaller by 2 Da, indicating the presence of a double bond in the smallest fragment (rings H and I). While no causative dinoflagellate has been identified at the sampling site, literature data on species isolated in the Mediterranean Sea and known producers of AZA analogs point to *A. poporum* as the dinoflagellate responsible for this toxic episode. The contribution of the recorded analogs to overall toxicity is discussed based on their estimated presence in the samples and literature data on their toxic potential. It is suggested that assessment of sample toxicity may involve quantitation of more than the three AZAs included to date.

## Figures and Tables

**Table 1:** Exact mass data of detected analogs.

Name	Exact Mass	Elemental Composition	Δ, ppm *	RT, min
AZA-2	856.5205	C_48_H_74_NO_12_	−0.7	5.78
AZA-6	842.5051	C_47_H_72_NO_12_	−0.5	4.83
AZA-9	858.4967	C_47_H_72_NO_13_	−4.3	3.56
AZA-10	858.5021	C_47_H_72_NO_13_	2.0	3.91
AZA-19	886.4949	C_48_H_72_NO_14_	−0.5	4.09
AZA-41	854.5044	C_48_H_72_NO_12_	−0.7	5.36
AZA-43	828.4894	C_46_H_70_NO_12_	0.2	4.63
884.5	884.4928	C_48_H_70_NO_14_	15.5	3.89

* Δ, ppm—difference between measured and calculated mass in parts per million.

**Table 2:** AZA levels detected during the toxic episode, expressed as a ratio (%) between the peak area of the analog and that of AZA-2, obtained by extracted ion chromatograms (XIC).

Date and Station	AZA-2 XIC Area	Analog XIC Area Relative to AZA-2 (%)						
	AZA-2	AZA-6	AZA-9	AZA-10	AZA-19	AZA-41	AZA-43	884.5
2 January 2024 S1	1.30 × 10^5^	56.50	27.4	18.1	73.5	41.6	52.7	26.0
2 January 2024 S2	3.86 × 10^5^	29.20	13.7	8.9	32.8	60.8	51.5	11.1
2 January 2024 S3	4.30 × 10^5^	36.50	17.7	10,8	35.2	3.2	52.6	15.8
7 January 2024 S1	3.51 × 10^5^	19.90	12.5	7.9	25.2	65.1	50.5	6.0
7 January 2024 S2	6.04 × 10^5^	15.50	9.2	6.4	24.3	62.5	46.7	7.9
7 January 2024 S3	1.68 × 10^5^	28.40	13.7	10.1	42.6	60.4	48.6	9.5
10 January 2024 S1	2.26 × 10^5^	22.00	27.6	10.6	42.4	55.8	48.1	13.4
10 January 2024 S2	1.55 × 10^5^	15.30	17.5	7.6	52.8	77.2	69.9	22.6
10 January 2024 S3	1.46 × 10^5^	18.80	22.3	8.7	52.2	75.5	61.1	21.1
24 January 2024 S1	-	-	-	-	-	-	-	-
24 January 2024 S2	-	-	-	-	-	-	-	-
24 January 2024 S3	-	-	-	-	-	-	-	-
29 January 2024 S1	6336.45	130.30	-	-	-	-	-	-
29 January 2024 S2	16,014.53	60.30	-	-	-	84.6	-	-
29 January 2024 S3	5318.2	146.60	-	-	99.3	-	-	-
5 February 2024 S1	10,289	60.40	-	-	137.3	-	-	-
5 February 2024 S2	16,049.5	32.90	-	-	-	-	-	-
5 February 2024 S3	16,783.49	35.90	-	-	59.7	-	-	-
14 February 2024 S1	3128.26	174.70	-	-	241.0	-	-	-
14 February 2024 S2	3533.74	95.90	-	-	186.4	-	-	-
14 February 2024 S3	11,010.7	28.30	-	-	56.5	-	-	-
19 February 2024 S1	1597.98	150.90	-	-	137.9	-	-	-
19 February 2024 S2	1989.23	-	-	-	-	-	-	-
19 February 2024 S3	1759.86	-	-	-	-	-	-	-
10 April 2024 S1	7349.2	100.60	-	-	-	-	-	-
10 April 2024 S2	7963.33	86.30	-	-	-	-	-	-
10 April 2024 S3	8438.44	92.70	-	-	-	-	-	-
15 April 2024 S1	5562.55	-	-	-	-	-	-	-
15 April 2024 S2	7380.33	-	-	-	-	-	-	-
15 April 2024 S3	7141.97	-	-	-	-	-	-	-
24 April 2024 S1	7174.9	-	-	-	-	-	-	-
24 April 2024 S2	12,457.55	-	-	-	-	-	-	-
24 April 2024 S3	7901.95	-	-	-	-	-	-	-
28 April 2024 S1	5572.41	-	-	-	-	-	-	-
28 April 2024 S2	10,633.13	-	-	-	-	-	-	-
28 April 2024 S3	6835.4	-	-	-	-	-	-	-
6 May 2024 S1	3958.01	-	-	-	-	-	-	-
6 May 2024 S2	-	-	-	-	-	-	-	-
6 May 2024 S3	-	-	-	-	-	-	-	-
20 May 2024 S1	-	-	-	-	-	-	-	-
20 May 2024 S2	-	-	-	-	-	-	-	-
20 May 2024 S3	-	-	-	-	-	-	-	-

**Table 3:** Precursor ions, product ions, and method parameters for quantification of regulated AZAs.

Compound Name	Precursor Ion (*m*/*z*)	Product Ions (*m*/*z*)	Ion Type	Collision Energy (eV)	Fragmentor Voltage (V)
AZA-1	842.5	824.5; 806.5	[M + H]^+^	30; 42	230
AZA-2	856.5	838.5; 820.5	[M + H]^+^	30;42	210
AZA-3	828.5	810.5; 792.5	[M + H]^+^	28;42	210

## Data Availability

Dataset available upon request from the authors.

## Supplementary Materials

The following supporting information can be downloaded at: https://www.mdpi.com/article/10.3390/molecules31010060/s1, Figure S1: Mass spectrum of AZA-2 (*m*/*z* 856.5) detected at a retention time of 5.78 min; Figure S2: Mass spectrum of AZA-6 (*m*/*z* 842.5) detected in shellfish samples at a retention time of 4.83 min; Figure S3A. Mass spectrum of AZA-9 (*m*/*z* 858.5) detected in shellfish samples at a retention time of 3.58 min; Figure S3B. Mass spectrum of AZA-9 (*m*/*z* 858.5) detected in shellfish samples at a retention time of 3.58 min, enlarged portion of a graph from *m*/*z* 750 to *m*/*z* 920; Figure S3C. Mass spectrum of AZA-9 (*m*/*z* 858.5) detected in shellfish samples at a retention time of 3.58 min, enlarged portion of a graph from *m*/*z* 600 to *m*/*z* 720; Figure S3D. Mass spectrum of AZA-9 (*m*/*z* 858.5) detected in shellfish samples at a retention time of 3.58 min, enlarged portion of a graph from *m*/*z* 330 to *m*/*z* 400; Figure S4: Mass spectrum of AZA-10 (*m*/*z* 858.5) detected at a retention time of 3.91 min; Figure S5: Mass spectrum of AZA-19 (*m*/*z* 886.5) detected at a retention time of 4.09 min; Figure S6: Mass spectrum of AZA-41 (*m*/*z* 854.5) detected at a retention time of 5.36 min; Figure S7: Mass spectrum of AZA-43 (*m*/*z* 828.5) detected at a retention time of 4.63 min; Figure S8A: Mass spectrum of a novel azaspiracid analog (*m*/*z* 884.5) detected at a retention time of 3.89 min; Figure S8B. Mass spectrum of a novel azaspiracid analog (*m*/*z* 884.5) detected in shellfish samples at a retention time of 3.58 min, enlarged portion of a graph from *m*/*z* 750 to *m*/*z* 900; Figure S8C. Mass spectrum of a novel azaspiracid analog (*m*/*z* 884.5) detected in shellfish samples at a retention time of 3.58 min, enlarged portion of a graph from *m*/*z* 130 to *m*/*z* 370; Figure S9. (a) Fragmentation of an AZA-41 analog with two 13C atoms in a molecule resembling fragmentation of an AZA-2, (b) Fragmentation of an AZA-2 analog with two 13C atoms in a molecule resembling fragmentation of an AZA-10. Insets presenting part of spectrum with enlarged three peak clusters typical for fragmentation of a molecule with two 13C atoms; Table S1: Elemental composition, theoretical *m*/*z*, measured *m*/*z*, and deviation from theoretical value of all detected AZA precursors and their fragments.

## Abbreviations

The following abbreviations are used in this manuscript:

AZAs	Azaspiracids
AZA-2	Azaspiracid-2
AZA-6	Azaspiracid-6
AZA-9	Azaspiracid-9
AZA-10	Azaspiracid-10
AZA-19	Azaspiracid-19
AZA-41	Azaspiracid-41
AZA-43	Azaspiracid-43
NMR	Nuclear Magnetic Resonance
eq.	Equivalents
DSP	Diarrheic Shellfish Poisoning
XIC	Extracted Ion Chromatograms
S1	Station 1
S2	Station 2
S3	Station 3
LC-MS/MS	Liquid Chromatography–Tandem Mass Spectrometry
MeOH	Methanol
ACN	Acetonitrile
FA	Formic Acid
AF	Ammonium Formate
LOD	Limit of Detection
LOQ	Limit of Quantification
S/N	Signal to Noise
CRMs	Certified Reference Materials
FDMT1	Freeze-Dried Mussel Tissue
RT	Retention Time
UHPLC	Ultra-High-Performance Liquid Chromatography
HPLC	High-Performance Liquid Chromatography
ESI	Electrospray Ionisation
TOF-MS	Time-of-Flight Mass Spectrometry
SRM^HR^	Selected Reaction Monitoring High-Resolution
ISVF	Ion Spray Voltage Front
GS1	Gas 1
GS2	Gas 2
CE	Collision Energy
DP	Declustering Potential
EFSA	European Food Safety Authority
FDA	Food and Drug Administration
TEFs	Toxicity Equivalency Factors
