# Supplementary material for: AZA Toxin Profiles by LC-HRMS in Shellfish from Šibenik Bay: AZA-2 Predominant Analog
Source: Molecules. 2025 Dec 23;31(1):60. doi: 10.3390/molecules31010060 (PMC12786966; doi:10.3390/molecules31010060)
Supplement: Supplementary file 1 [file molecules-31-00060-s001.zip › molecules-3985566-supplementary.pdf]

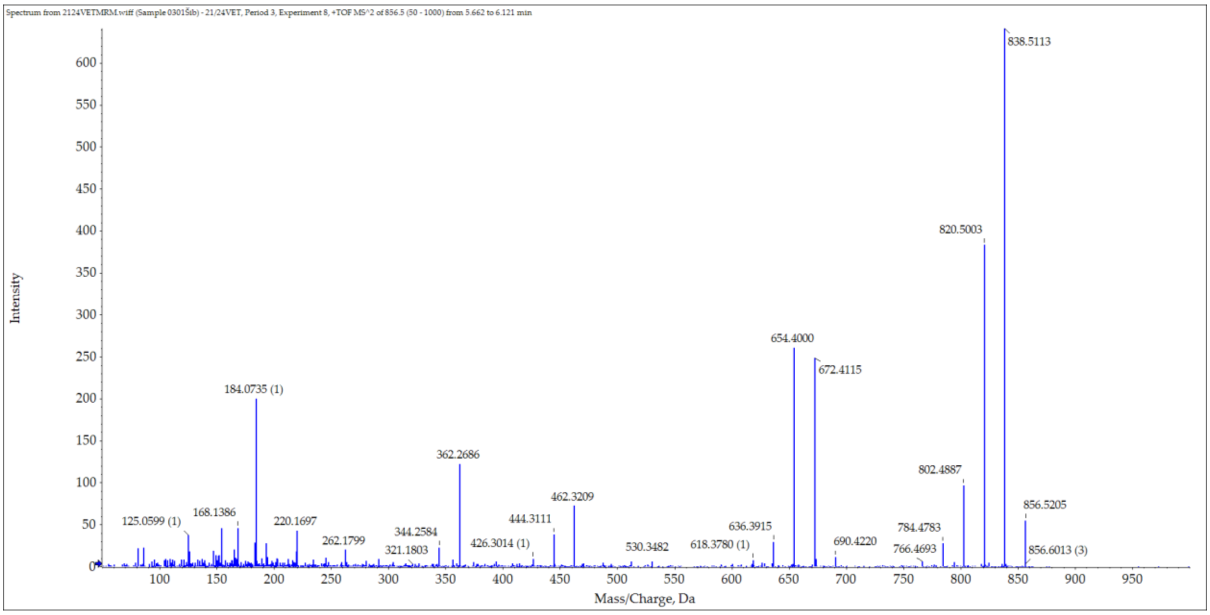

Figure S1: Mass spectrum of AZA-2 (*m/z* 856.5) detected at a retention time of 5.78 min.

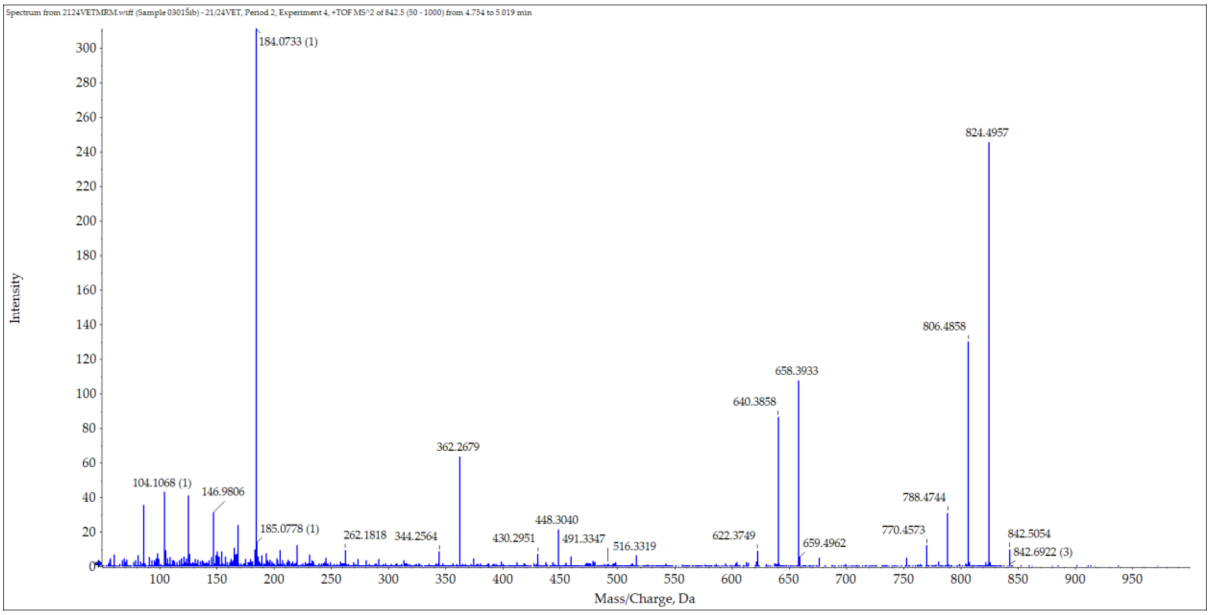

Figure S2: Mass spectrum of AZA-6 (*m/z* 842.5) detected in shellfish samples at a retention time of 4.83 min.

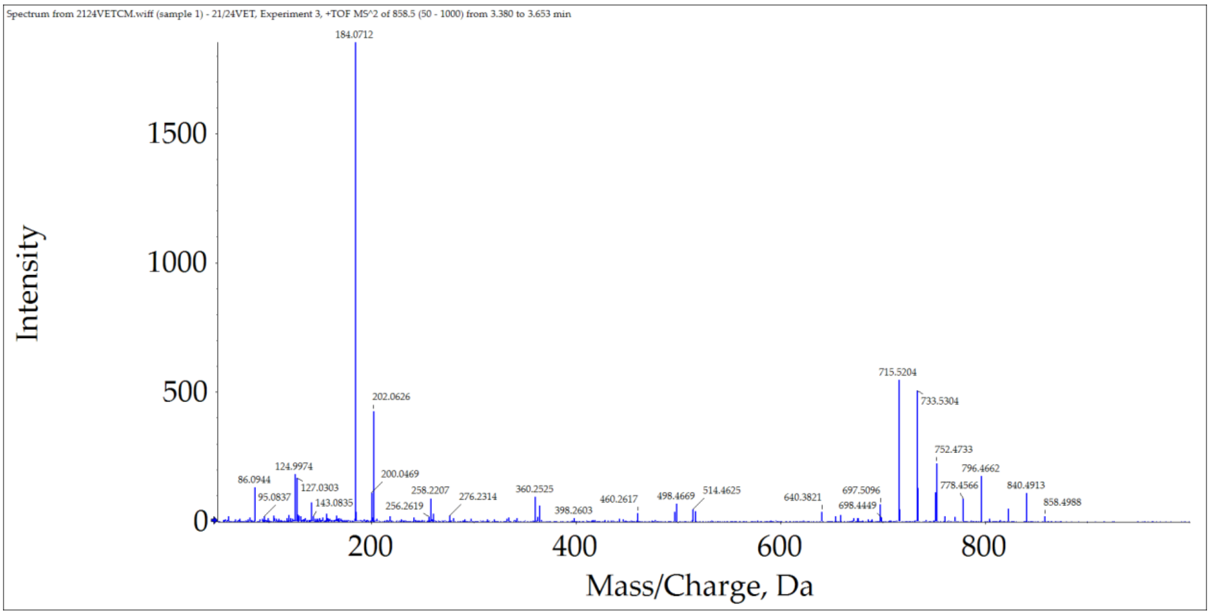

Figure S3A. Mass spectrum of AZA-9 (*m/z* 858.5) detected in shellfish samples at a retention time of 3.58 minutes.

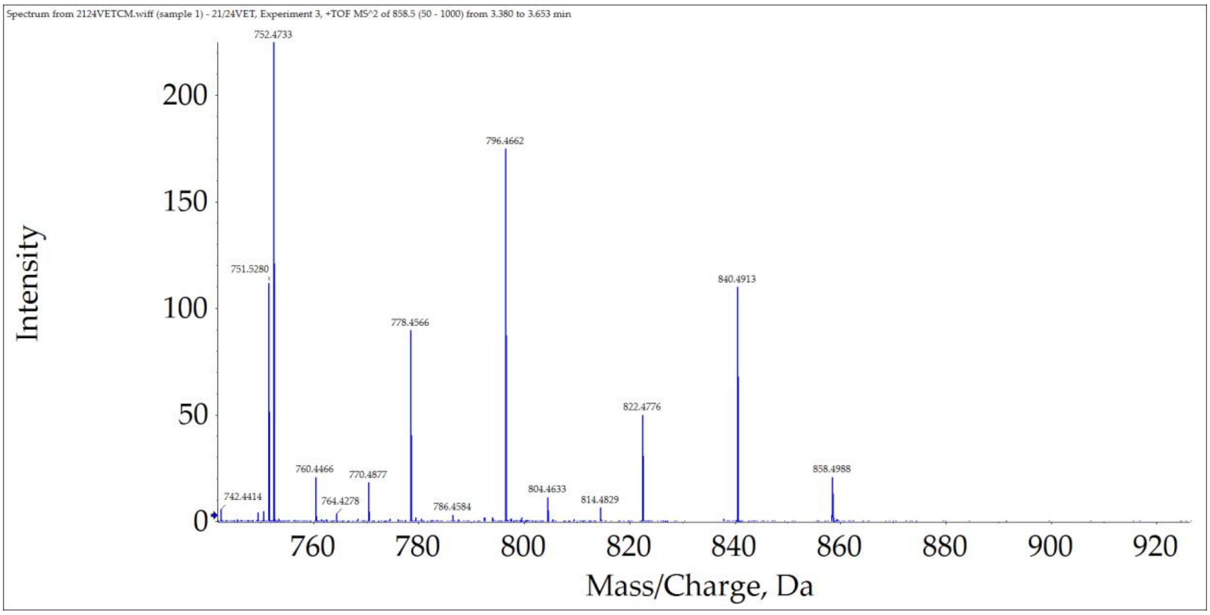

Figure S3B. Mass spectrum of AZA-9 (*m/z* 858.5) detected in shellfish samples at a retention time of 3.58 minutes, enlarged portion of a graph from *m/z* 750 to *m/z* 920

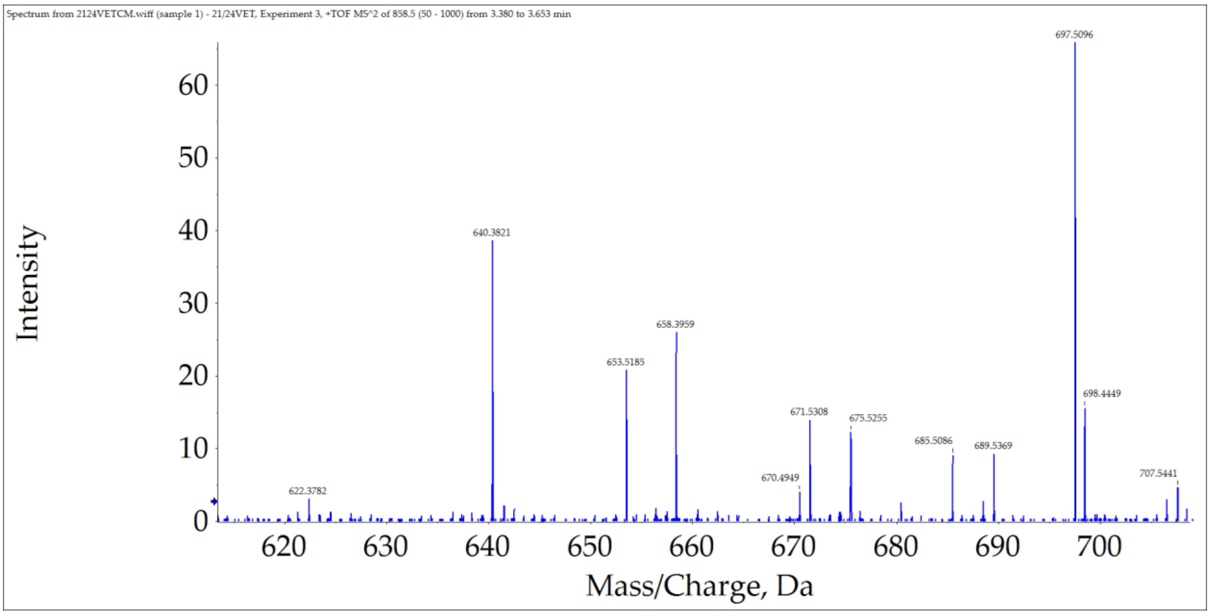

Figure S3C. Mass spectrum of AZA-9 (*m/z* 858.5) detected in shellfish samples at a retention time of 3.58 minutes, enlarged portion of a graph from m/z 600 to m/z 720

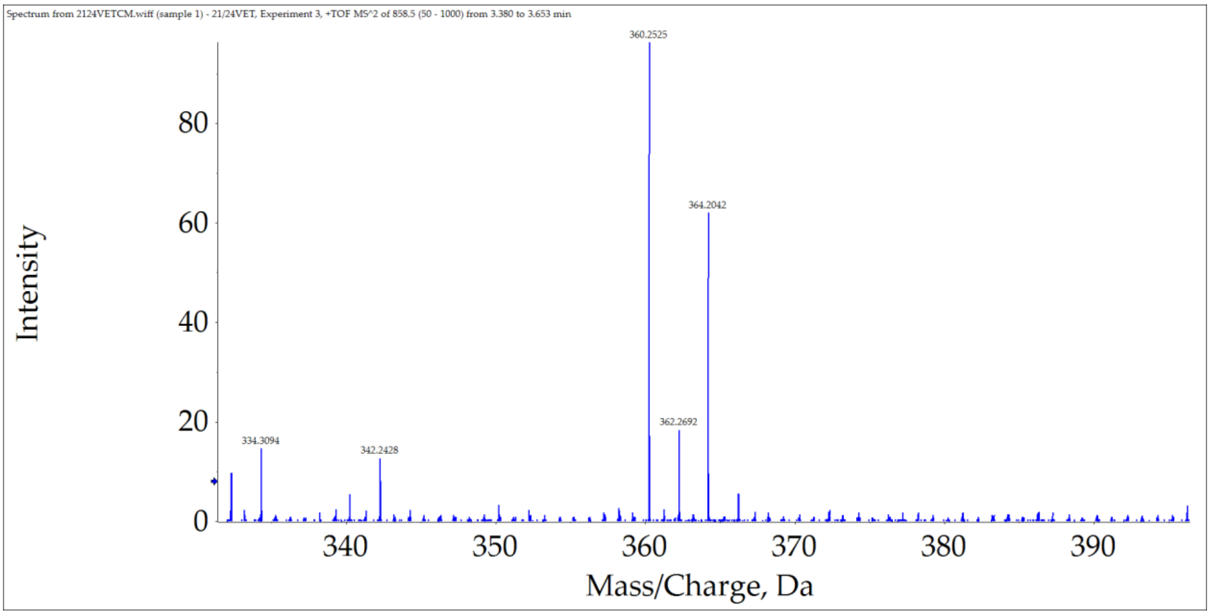

Figure S3D. Mass spectrum of AZA-9 (*m/z* 858.5) detected in shellfish samples at a retention time of 3.58 minutes, enlarged portion of a graph from m/z 330 to m/z 400

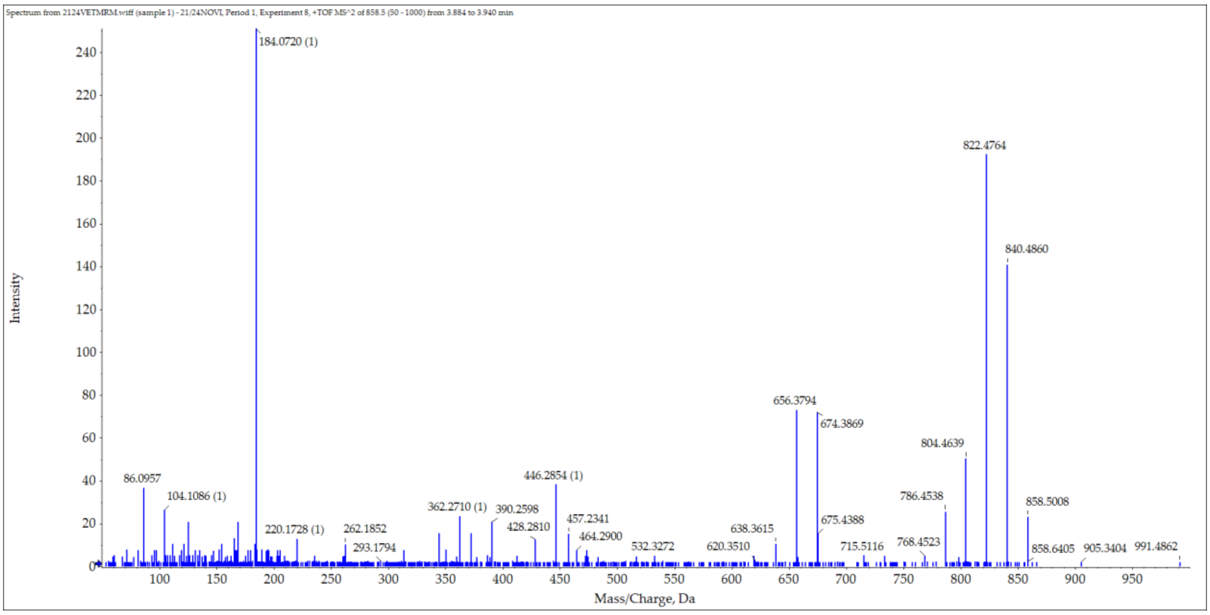

Figure S4: Mass spectrum of AZA-10 (*m/z* 858.5) detected at a retention time of 3.91 minutes.

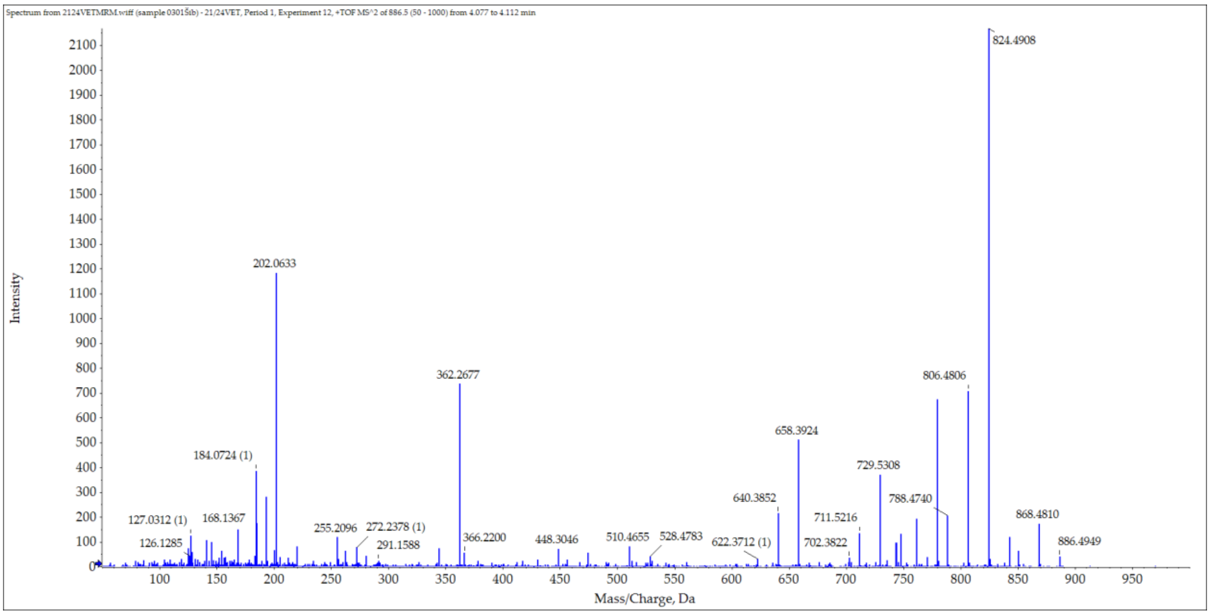

Figure S5: Mass spectrum of AZA-19 (*m/z* 886.5) detected at a retention time of 4.09 min

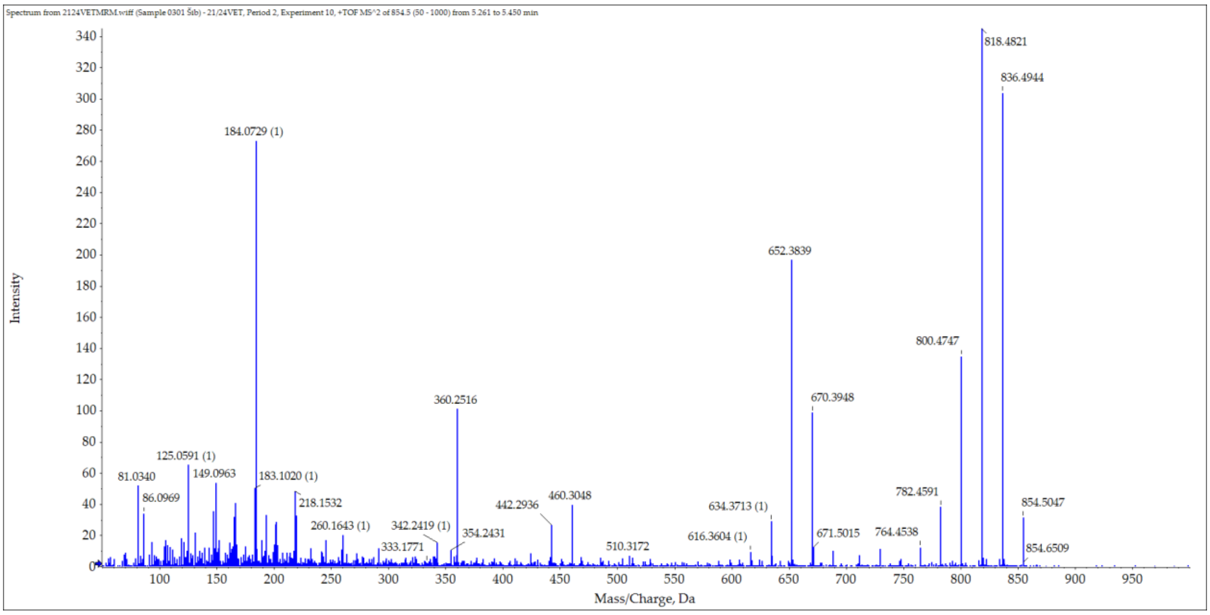

Figure S6: Mass spectrum of AZA-41 (*m/z* 854.5) detected at a retention time of 5.36 minutes.

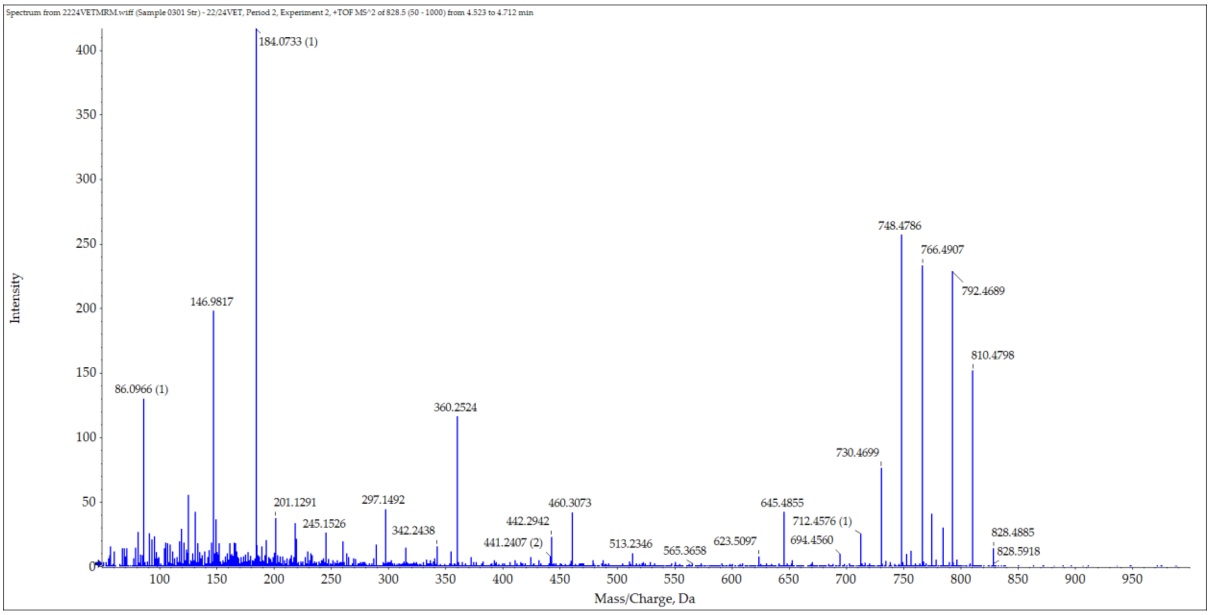

Figure S7: Mass spectrum of AZA-43 (*m/z* 828.5) detected at a retention time of 4.63 min.

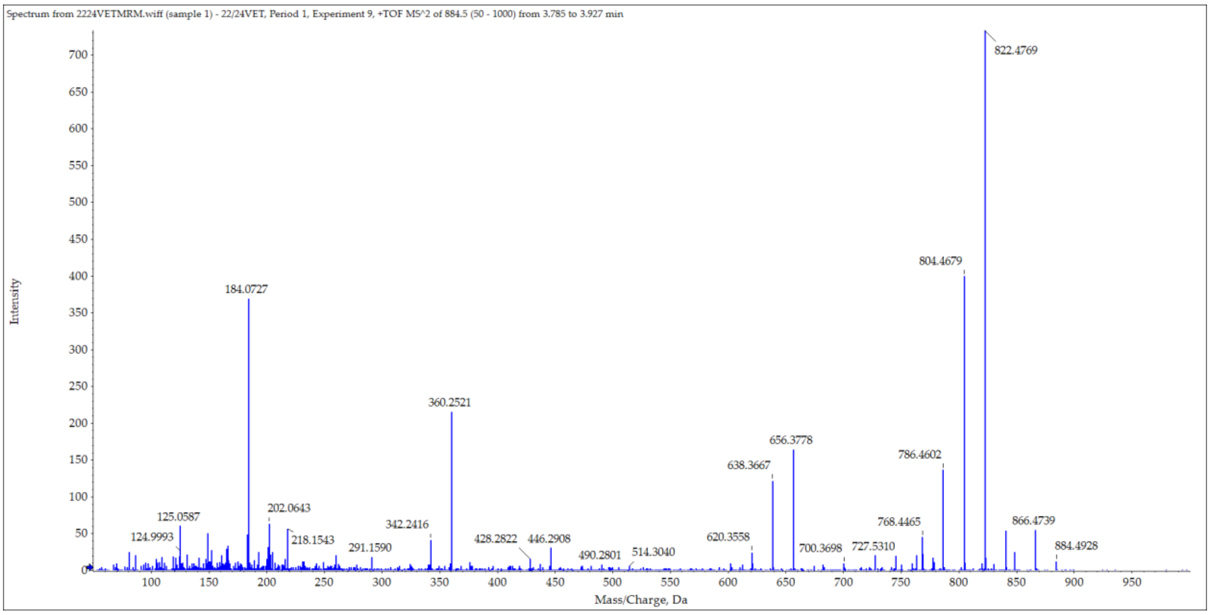

Figure S8A: Mass spectrum of a novel azaspiracid analog (m/z 884.5) detected at a retention time of 3.89 min.

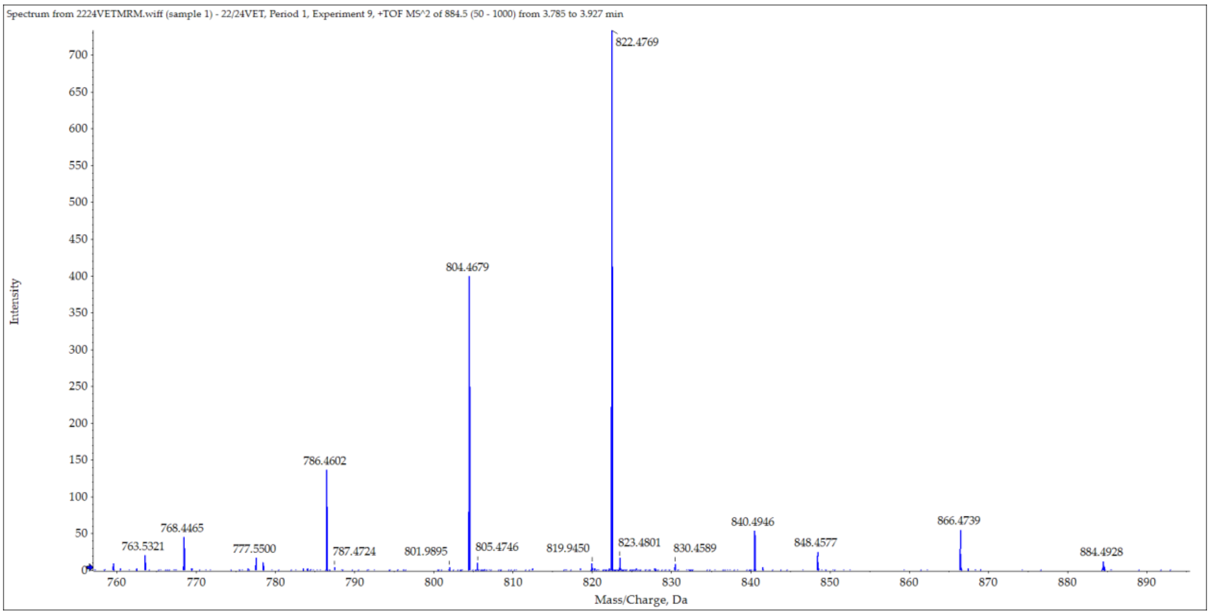

Figure S8B. Mass spectrum of a novel azaspiracid analog (m/z 884.5) detected in shellfish samples at a retention time of 3.58 minutes, enlarged portion of a graph from *m/z* 750 to *m/z* 900

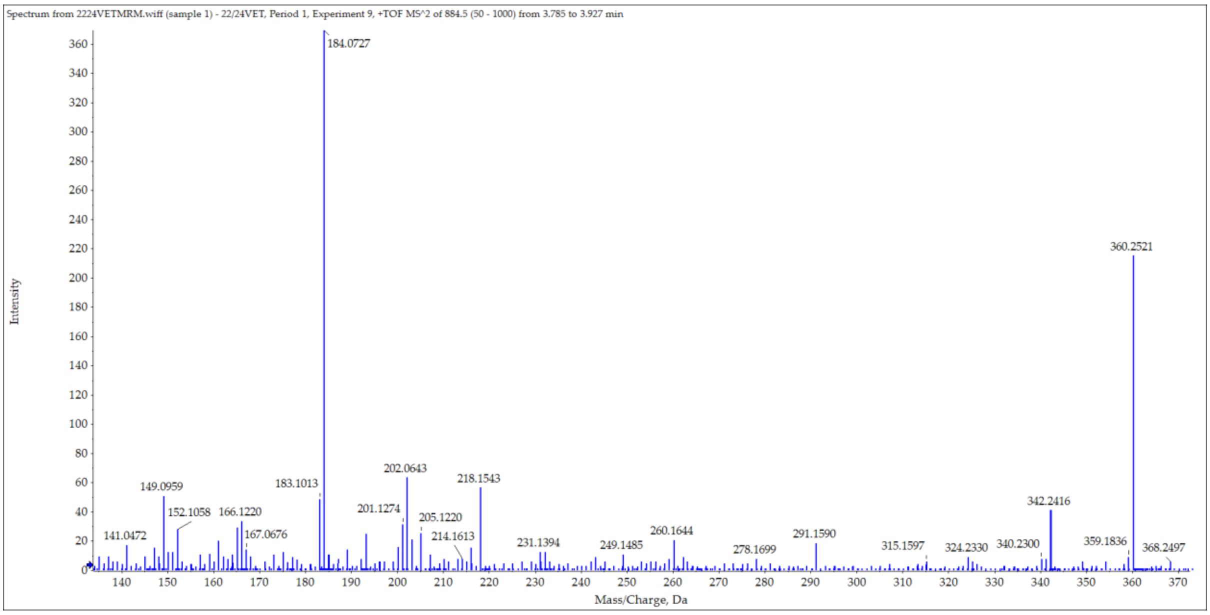

Figure S8C. Mass spectrum of a novel azaspiracid analog (m/z 884.5) detected in shellfish samples at a retention time of 3.58 minutes, enlarged portion of a graph from *m/z* 130 to *m/z* 370

Table S1. Elemental composition, theoretical *m/z,* measured *m/z*, and deviation from theoretical value of all detected AZA precursors and their fragments

AZA-2					
**Ion**		**Formula**	**Theoretical m/z**	**Measured m/z**	**Δ ppm**
Precursor		C48H74NO12	856.5205	856.5204	-0.2
Group 1	-H_2O	C48H72NO11	838.5060	838.5113	1.6
	-2H_2O	C48H70NO10	820.4994	820.5004	1.2
	-3H_2O	C48H68NO9	802.4889	802.4887	-0.2
Group 2	-H_2O	C38H58NO9	672.4106	672.4115	1.3
	-2H_2O	C38H56NO8	654.4000	654.3999	-0.2
Group 3	-H_2O	C27H44NO5	462.3214	462.3208	-1.3
Group 4	-H_2O	C22H36NO3	362.2690	362.2686	-1.0
Group 5	-H_2O	C16H24NO2	262.1801	262.1800	-0.6
Group 6	-H_2O	C10H18NO	168.1383	168.1386	1.8
**AZA-6**					
**Ion**		**Formula**	**Theoretical m/z**	**Measured m/z**	**Δ ppm**
Precursor		C47H72NO12	842.5055	842.5051	-0.7
Group 1	-H_2O	C47H70NO11	824.4943	824.4940	-0.4
	-2H_2O	C47H68NO10	806.4838	806.4861	2.9
	-3H_2O	C47H66NO9	788.47321	788.4744	1.5
Group 2	-H_2O	C37H56NO9	658.3950	658.3931	-2.8
	-2H_2O	C37H54NO8	640.3844	640.3858	2.2
	-3H_2O	C37H52NO7	622.3738	622.3749	1.7
Group 3	-H_2O	C26H42NO5	448.3057	448.3040	-3.9
Group 4	-H_2O	C22H36NO3	362.2690	362.2678	-3.2
Group 5	-H_2O	C16H24NO2	262.1802	262.1818	6.3
Group 6	-H_2O	C10H18NO	168.1383	-	-
**AZA-9**					
**Ion**		**Formula**	**Theoretical m/z**	**Measured m/z**	**Δ ppm**
Precursor		C47H72NO13	858.4998	858.4988	-1.2
Group 1	-H_2O	C47H70NO12	840.4892	840.4913	2.5
	-2H_2O	C47H68NO11	822.4787	822.4776	-1.4
Group 2	-H_2O	C37H56NO9	658.3950	658.3959	1.4
	-2H_2O	C37H54NO8	640.3843	640.3821	-3.5
Group 3	-	-	-	-	-
Group 4	-H_2O	C22H36NO3	362.2690	362.2692	0.7
Group 5	-	-	-	-	-
Group 6	-	-	-	-	-
**AZA-10**					
**Ion**		**Formula**	**Theoretical m/z**	**Measured m/z**	**Δ ppm**
Precursor		C47H72NO13	858.4998	858.5008	1.2
Group 1	-H_2O	C47H70NO12	840.4892	840.4860	-3.8
	-2H_2O	C47H68NO11	822.4787	822.4764	-2.8
Group 2	-H_2O	C37H56NO10	674.3899	674.3869	-4.4
	-2H_2O	C37H54NO9	656.3793	658.3794	0.1

	-3H_2O	C37H52NO8	638.3687	638.3615	-11.3
Group 3	-H_2O	C26H42NO6	464.3006	464.2900	-23.0
	-2H_2O	C26H40NO5	446.2901	446.2854	-10.5
	-3H_2O	C26H38NO4	428.2795	428.2810	3.4
Group 4	-H_2O	C22H36NO3	362.2690	362.2710	5.5
Group 5	-H_2O	C16H24NO2	262.1802	262.1852	19.1
Group 6	-	-	-	-	-
**AZA-19**					
**Ion**		**Formula**	**Theoretical m/z**	**Measured m/z**	**Δ ppm**
Precursor		C48H72NO14	886.4947	886.4949	0.2
Group 1	-H_2O	C48H70NO13	868.4842	868.4810	-3.7
	-2H_2O	C48H68NO12	850.4736	-	-
	-H_2O-CO_2	C47H70NO11	824.4943	824.4908	-4.3
	-2H_2O-CO_2	C47H68NO10	806.4838	806.4806	-3.9
Group 2	-H_2O	C38H56NO11	702.3848	702.3822	-3.7
	-2H_2O	C38H54NO10	684.3742	-	-
	-H_2O-CO_2	C37H56NO9	658.3950	658.3924	-3.9
	-2H_2O-CO_2	C37H54NO8	640.3840	640.3852	1.3
Group 3	-H_2O	C27H42NO7	492.2956	-	-
	-2H_2O	C27H40NO6	490.2799	-	-
	-3H_2O	C27H38NO5	488.2643	-	-
Group 4	-H_2O	C22H36NO3	362.2690	362.2677	-3.6
Group 5	-H_2O	C16H24NO2	262.1802	-	-
Group 5	-H_2O	C10H18NO	168.1383	168.1367	-9.5
**AZA-41**					
**Ion**		**Formula**	**Theoretical m/z**	**Measured m/z**	**Δ ppm**
Precursor		C48H72NO12	854.5049	854.5047	-0.2
Group 1	-H_2O	C48H70NO11	836.4943	836.4944	0.1
	-2H_2O	C48H68NO10	818.4838	818.4821	-2.0
	-3H_2O	C48H66NO9	800.4732	800.4747	1.9
Group 2	-H_2O	C38H56NO9	670.3950	670.3947	-0.2
	-2H_2O	C38H54NO8	652.3844	652.3839	-0.8
	-3H_2O	C38H52NO7	634.3738	634.3713	-4.0
Group 3	-H_2O	C27H42NO5	460.3057	460.3048	-2.1
	-2H_2O	C27H40NO4	442.2952	442.2932	-4.5
Group 4	-H_2O	C22H34NO3	360.2533	360.2516	-4.8
	-2H_2O	C22H32NO2	342.2428	342.2419	-2.5
Group 5	-H_2O	C16H22NO2	260.1645	260.1643	-0.8
Group 6	-H_2O	C10H16NO	166.1226	166.1240	8.2
**AZA-43**					
**Ion**		**Formula**	**Theoretical m/z**	**Measured m/z**	**Δ ppm**
Precursor		C46H70NO12	828.4892	828.4894	0.2
Group 1	-H_2O	C46H68NO11	810.4787	810.4798	1.4
	-2H_2O	C46H66NO10	792.4681	792.4689	1.0
	-3H_2O	C46H64NO9	774.4576	-	-
	-CO_2	C45H70NO10	784.4994	-	-
	-H_2O-CO_2	C45H68NO9	766.4889	766.4907	2.4

	$-2H_2O-CO_2$	C45H66NO8	748.4783	748.4786	0.4
	$-3H_2O-CO_2$	C45H64NO7	730.4677	730.4699	3.0
Group 2	-	-	-	-	-
Group 3	$-H_2O$	C27H42NO5	460.3057	460.3073	3.4
	$-2H_2O$	C27H40NO4	442.2952	442.2942	-2.2
Group 4	$-H_2O$	C22H34NO3	360.2533	360.2524	-2.6
Group 5	$-H_2O$	C16H22NO2	260.1645	-	-
Group 6	$-H_2O$	C10H16NO	166.1226	-	-
**884**					
**Ion**		**Formula**	**Theoretical m/z**	**Measured m/z**	**Δ ppm**
Precursor		C48H70NO14	884.4791	884.4928	15.5
Group 1	$-H_2O$	C48H68NO13	866.4685	866.4739	6.3
	$-2H_2O$	C48H66NO12	848.4579	848.4577	-0.3
	$-3H_2O$	C48H64NO11	830.4474	-	-
	$-CO_2$	C47H70NO12	840.4892	840.4946	6.4
	$-H_2O-CO_2$	C47H68NO11	822.4787	822.4769	-2.2
	$-2H_2O-CO_2$	C47H66NO10	804.4681	804.4679	-0.2
	$-3H_2O-CO_2$	C47H64NO9	786.4576	786.4602	2.6
Group 2	$-H_2O$	C38H54NO11	700.3691	700.3698	1.0
	$-2H_2O$	C38H52NO10	682.3586	-	-
	$-H_2O-CO_2$	C37H54NO9	656.3793	656.3778	-2.3
	$-2H_2O-CO_2$	C37H52NO8	638.3687	638.3677	-1.6
	$-3H_2O-CO_2$	C37H50NO7	620.3582	620.3558	-3.8
Group 3	$-H_2O$	C27H40NO7	490.2799	490.2801	0.4
	$- H_2O-CO_2$	C26H40NO5	446.2901	446.2908	1.5
Group 4	$-H_2O$	C22H34NO3	360.2533	360.2521	-3.4
	$-2H_2O$	C22H32NO2	342.2428	342.2416	-3.4
Group 5	$-H_2O$	C16H22NO2	260.1645	260.1644	-0.3
Group 6	$-H_2O$	C10H16NO	166.1226	166.1220	-3.6

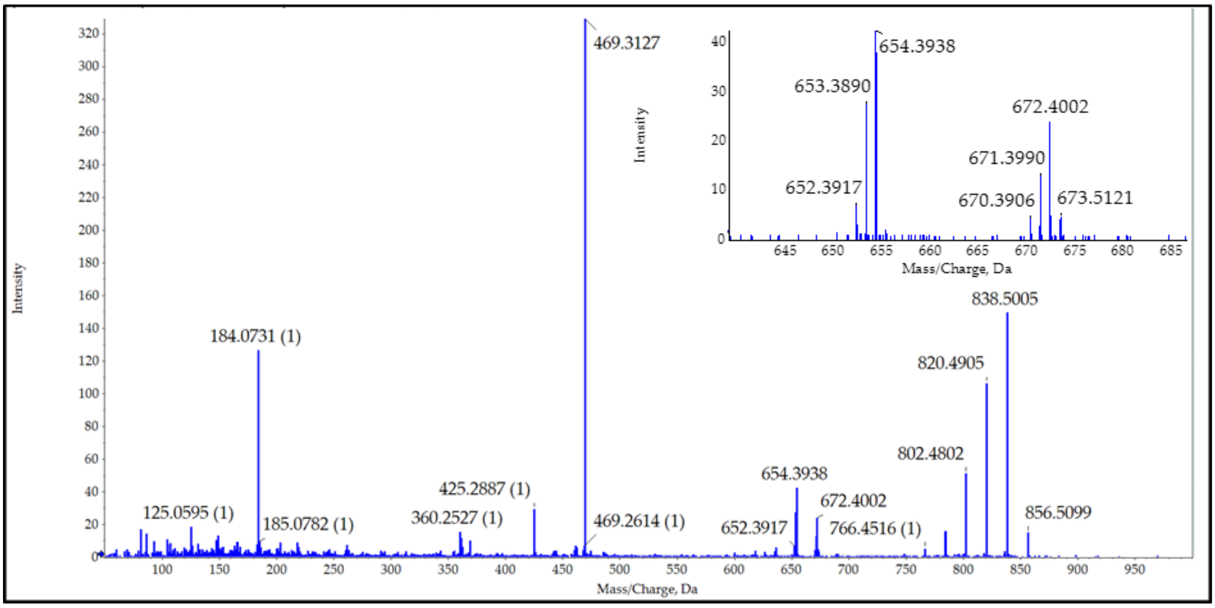

(a)

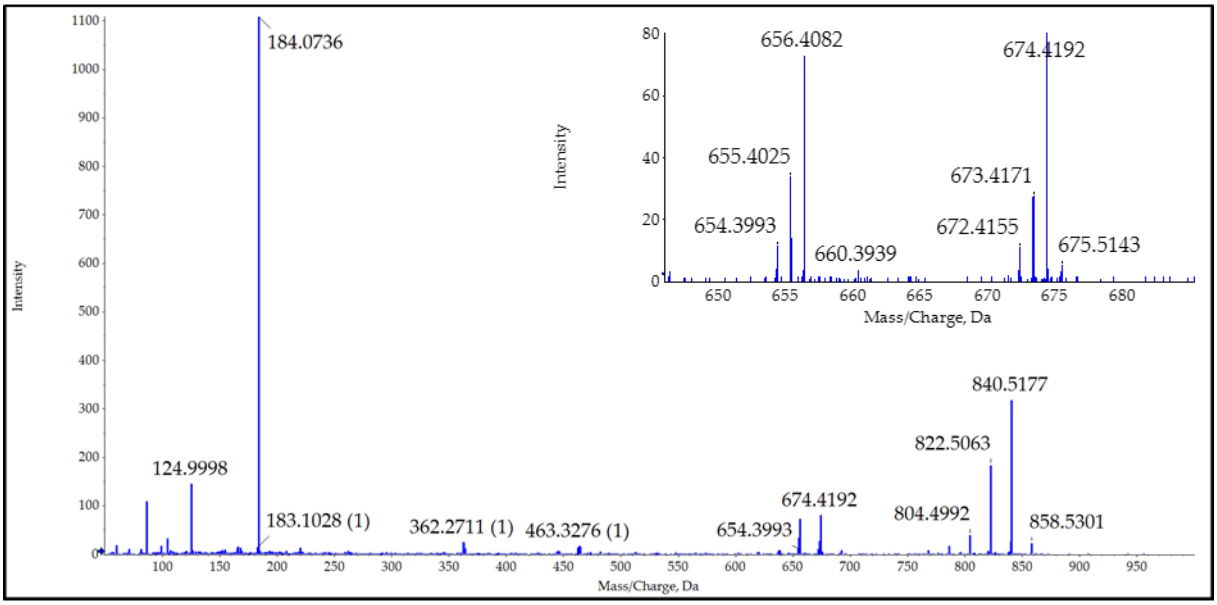

(b)

Figure S9. (a) - Fragmentation of an AZA-41 analog with two ^{13}C atoms in a molecule resembling fragmentation of an AZA-2, (b) - Fragmentation of an AZA-2 analog with two ^{13}C atoms in a molecule resembling fragmentation of an AZA-10. Insets presenting part of spectrum with enlarged three peak clusters typical for fragmentation of a molecule with two ^{13}C atoms.
